# IgM Augments Complement Bactericidal Activity with Serum from a Patient with a Novel *CD79a* Mutation

**DOI:** 10.1007/s10875-017-0474-7

**Published:** 2018-01-15

**Authors:** Jeroen D. Langereis, Stefanie S Henriet, Saskia Kuipers, Corry M.R. Weemaes, Mirjam van der Burg, Marien I. de Jonge, Michiel van der Flier

**Affiliations:** 1 Section Pediatric Infectious Diseases, Laboratory of Medical Immunology, Radboud Institute for Molecular Life Sciences, Radboud University Medical Center, Nijmegen, The Netherlands; 2 Pediatric Infectious Diseases and Immunology, Department of Pediatrics, Amalia Children’s Hospital, Radboud University Medical Center, Nijmegen, The Netherlands; 3 Medical Microbiology, Radboud University Medical Center, Nijmegen, The Netherlands; 4 Department of Immunology, Erasmus MC, University Medical Center Rotterdam, Rotterdam, The Netherlands

**Keywords:** Antibodies, IgM, bacterial, complement, immunodeficiency disease

## Abstract

Antibody replacement therapy for patients with antibody deficiencies contains only IgG. As a result, concurrent IgM and IgA deficiency present in a large proportion of antibody deficient patients persists. Especially patients with IgM deficiency remain at risk for recurrent infections of the gastrointestinal and respiratory tract. The lack of IgM in the current IgG replacement therapy is likely to contribute to the persistence of these mucosal infections because this antibody class is especially important for complement activation on the mucosal surface. We evaluated whether supplementation with IgM increased serum bactericidal capacity in vitro. Serum was collected from a patient with agammaglobulinemia and supplemented with purified serum IgM to normal levels. Antibody and complement deposition on the bacterial surface was determined by multi-color flow cytometry. Bacterial survival in serum was determined by colony-forming unit counts. We present a patient previously diagnosed with agammaglobulinemia due to *CD79A* (Igα) deficiency revealing a novel pathogenic insertion variant in the *CD79a* gene (NM_001783.3:c.353_354insT). Despite IgG replacement therapy and antibiotic prophylaxis, this patient developed a *Campylobacter jejuni* spondylodiscitis of lumbar vertebrae L4–L5. We found that serum IgM significantly contributes to complement activation on the bacterial surface of *C. jejuni*. Furthermore, supplementation of serum IgM augmented serum bactericidal activity significantly. In conclusion, supplementation of intravenous IgG replacement therapy with IgM may potentially offer greater protection against bacterial infections, also in the context of increasing antibiotic resistance.

## Introduction

Patients with agammaglobulinemia have very low or no serum immunoglobulin levels, making these patients highly susceptible to infections. The largest group of patients has X-linked agammaglobulinemia (XLA), which is a caused by a defect in the *Btk* gene encoding Bruton Tyrosine Kinsase (*Btk*), which accounts for 85% of agammaglobulinemia patients. A smaller group has defects in for instance *IGLL1*, *CD79A*, *CD79B*, or *BLNK*, which have clinical findings that are similar from those seen in patients with mutations in *Btk* but tend to have a more severe onset of disease [1].

Treatment relies on the use of IgG replacement therapy (IgGRT), sometimes in conjunction with prophylactic antibiotic use. IgGRT was first introduced in 1952 by Colonel Ogden Bruton and applied to the first patient with agammaglobulinemia [2]. IgGRT is still the main therapy to date and successfully reduces the frequency of invasive bacterial infections in patients with immunoglobulin deficiency [3]. However, despite IgGRT, agammaglobulinemia patients still have increased infection rates and lowered life expectancy [4]. Currently used immunoglobulin preparations contain only IgG. As a result, concurrent IgM and IgA deficiency present in a large proportion of antibody deficient patients persists. Especially patients with IgM deficiency, such as agammaglobulinemia patients, remain at risk for recurrent infections of the respiratory tract [5]. Gastrointestinal manifestations also present a significant burden; 35% of XLA patients suffer from complications including recurrent infections and inflammatory bowel disease [6]. The lack of especially IgM in the current IgGRT is likely to contribute to the persistence of these mucosal infections because this antibody class is especially important for complement activation on the mucosal surface.

Patients with selective IgM deficiency (sIgMD) represent another model disease where IgM is absent. The immunological and clinical phenotype of sIgMD is very heterogeneous, and patients can even remain asymptomatic [7]. In a series of 17 sIgMD patients, recurrent upper respiratory tract infections were observed in five out of six patients with undetectable IgM levels (< 0.05 g/L) [7]. Patients with sIgMD can also present with invasive manifestations such as sepsis and meningitis [8]. Although for most sIgMD patients, IgGRT was not required [7]; it is suggested to be beneficial for patients with recurrent or severe infections [9].

Previous observational work demonstrated an important protective effect of IgM against colonization of the respiratory tract by non-typeable *Haemophilus influenzae* (NTHi) in patients with hypogammaglobulinemia [10]. We showed that especially recognition of NTHi lipooligosaccharide by IgM efficiently increased complement- and neutrophil-mediated killing [11, 12], which might contribute to the protective effect of IgM against colonization [10]. We hypothesized that the superior efficacy of complement activation by IgM compared to IgG may explain this observation. This may be especially relevant for protection against mucosal Gram-negative bacteria.

Here, we describe a patient with a *CD79A* (Igα) deficiency due to compound heterozygous mutations in the *CD79a* gene. Igα is the signal transduction molecule of the pre-B cell receptor. Absence of Igα results in a block from pro-B cells to pre-B cells in the B cell differentiation pathway, resulting in agammaglobulinemia [1].

We show that IgM supplementation of serum from a *CD79A* deficient agammaglobulinemia patient on IgG substitution therapy increases serum bactericidal activity against *Campylobacter jejuni* in vitro. This finding suggests that IgM containing immunoglobulin preparations may improve protective efficacy compared to the current IgGRT.

## Material and Methods

### Ethics Statement

The study was approved by the ethics committee of the Radboudumc, Nijmegen, the Netherlands. Written informed consent was obtained from the patient. Collection of blood from healthy volunteers was approved by the ethics committee of the Radboudumc, Nijmegen, the Netherlands. All experiments were carried out in accordance with local guidelines and regulations and comply with the Declaration of Helsinki and the Good Clinical Practice guidelines.

### Serum Preparation and Storage

Blood was drawn in vacutainer gel serum tubes (BD Biosciences) and kept on ice within 30 min following collection. Serum was prepared by centrifuging blood for 10 min at 4000×*g*, and serum aliquots were stored at − 80 °C.

Serum was diluted in HBSS without phenol red containing Ca^2+^ and Mg^2+^ + 0.1% gelatin (Hepes3+). For complement-inactivation, serum was heat-inactivated (HI) by incubating 20 min at 56 °C. Purified IgM from human serum (Sigma-Aldrich, I8260) was washed with PBS on a Amicon Ultra-0.5 Centrifugal Filter Unit column (Millipore) to remove the preservative sodium azide and suspended into PBS at a concentration of 1 mg/mL.

### Bacterial Growth Conditions and Storage

The patient’s *C. jejuni* isolate was used for in vitro functional assays. Bacteria were grown on blood agar plates in a microaerobic environment at 42 °C. Bacteria were harvested directly from plate and suspended into PBS containing 16% glycerol for storage.

### Flow Cytometry

Bacteria were washed with Hepes3+ and diluted to an OD_620_ of 0.1. For IgG, IgM, C3, and C5b9 surface opsonization, 50 μL bacteria were incubated with 50 μL 10% serum in Hepes3+ for 15 min at 37 °C. Opsonized bacteria were washed, fixed for 20 min with 2% paraformaldehyde, and incubated with 1:500-diluted C3-specific polyclonal antibody (MP Biomedical, Cappel), 1:100-diluted C5b-9-specific monoclonal antibody (Santa Cruz biotechnology), 1:50-diluted Fcγ Fragment Specific PerCP AffiniPure Goat Anti-Human IgG (Jackson Immunoresearch), and 1:500-diluted Fc5μ fragment specific Alexa Fluor® 647 AffiniPure Goat Anti-Human IgM (Jackson Immunoresearch) in PBS with 2% BSA for 15 min at room temperature. Bacteria were washed and incubated with 1:200 PE-labeled goat anti-mouse IgG (ThermoFisher Scientific) in PBS with 2% BSA for 15 min at room temperature. Bacteria were washed and suspended in PBS for flow cytometry using a FACS LSR II instrument (BD Biosciences). Data were analyzed using the software program FlowJo version 10.2. Data is presented as geometric mean fluorescence intensity (MFI) in arbitrary units (AU).

### Bacterial Survival Assays

Bacteria were washed with Hepes3+ and diluted to a concentration of ∼ 20.000 colony forming units (CFU)/mL. Fifty microliter of bacteria was mixed with 50 μL serum or HI-serum diluted in Hepes3+ and incubated 30 min at 37 °C. Serial dilutions were plated on blood agar plates and incubated in a candle jar at 42 °C. Survival was determined by dividing the CFU counts in serum with the CFU count in HI-serum.

## Results

### Case Report Campylobacter Jejuni Spondylodiscitis in a Patient with Agammaglobulinemia Associated with Novel *CD79a* Pathogenic Insertion Variant

Here, we describe a 16-year-old male, who was diagnosed with agammaglobulinemia associated with *CD79a* deficiency at 2 years of age. Initially, he presented with severe bacterial pneumonia associated with nasopharyngeal carriage of *Haemophilus influenzae* type B. In addition, he suffered from chronic otitis media with effusion. Initial immunologic assessment demonstrated agammaglobulinemia (IgG < 0.5 g/L, IgA < 0.13 g/L, and IgM < 0.06 g/L). Immunophenotyping of peripheral blood revealed total absence of CD19^+^ B cells. Bone marrow aspirate showed decreased percentages of CD22+ B cells (20%, normal < 5 years of age 36 ± 7%), with absence of *CD79A* expression. Sequence analysis confirmed the diagnosis of *CD79a* deficiency due to compound heterozygous mutations: in one allele, we found the NM_001783.3:c.380-2A > G pathogenic variant as described previously [13]; in the second allele, we found a novel insertion in exon 2 of a thymine (c.353insT). This variation creates a frame shift starting at p.(Cys119Leufs*63). The new reading frame ends in a STOP codon 63 positions downstream. This is the sixth patient with *CD79a* mutations described to date.

After the diagnosis, subcutaneous IgGRT was started and combined with antibiotic prophylaxis. The patient received weekly subcutaneous IgG at a dose of 320 mg/kg/28d (Hizentra; CSL Behring AG, Swiss) (IgG through level 8–10 g/L) and was on prophylactic amoxicillin 500 mg orally twice daily. To facilitate travel during summer holidays, subcutaneous IgGRT was interrupted for maximally 3 weeks at a time and intravenous IgGRT at a dose of 365 mg/kg/28d was administered before traveling. The patient had a mild subsequent clinical course with episodes of gastrointestinal parasitic infections and chronic conjunctivitis.

At age 16, the patient presented with a 3-week history of lower back pain without fever. Laboratory evaluation demonstrated leukocytes of 12.10^9^/L, CRP 37 mg/L, and ESR 15 mm/h; MRI examination showed spondylodiscitis of lumbar vertebrae L4–L5. He was initially started on empiric flucloxacillin and gentamicin. Blood culture was positive for a *Campylobacter jejuni* strain, which was resistant for tetracycline (8 mg/L), but susceptible to erythromycin/azithromycin (4 mg/L) and ciprofloxacin (0.125 mg/L) according to the EUCAST criteria. The strain was additionally tested for amoxicillin (256 mg/L, resistant) and amoxicillin-clavulanic acid (2 mg/L, susceptible), which indicates the presence of beta-lactamase activity. The patient was switched to azithromycin 500 mg once daily and treated orally for 9 months in total with good clinical recovery. As preferred by the patient, IgGRT was switched from subcutaneous to intravenous at 500 mg/kg/28 days. Additional tests of complement function showed normal classical (CH50 102% (67–149%)) and alternative pathway (AP50 68% (67–133%)) activity. Since patients with agammaglobulinemia receiving IgGRT only have corrected IgG serum levels, we evaluated whether supplementation with serum IgM increased serum bactericidal capacity in vitro.

### Campylobacter Jejuni-Specific IgG at Different IgG Concentrations

Six months previous to presentation with spondylodiscitis, subcutaneous IgGRT was briefly interrupted for summer holidays, while the patient was protected with an equal dose intravenous IgG. Serum samples were taken before and after intravenous IgG administration as part of a research protocol. IgG, IgA, and IgM serum levels were determined, and IgG concentration was 8.08 g/L pre-intravenous IgG, and 14.8 g/L post-intravenous IgG, but both samples were within normal range (5.2–15.6 g/L), whereas IgA and IgM concentrations were below the detection limit in both samples (< 0.07 g/L).

**Fig. 1:**
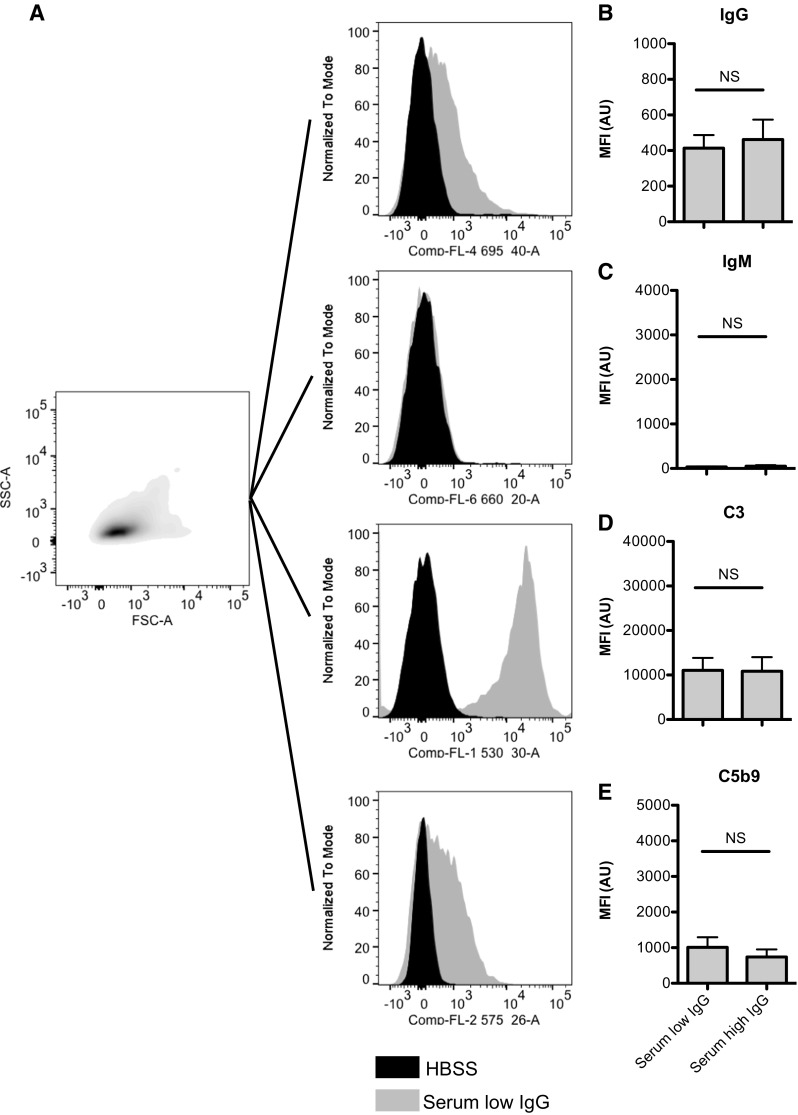
*Similar recognition of Campylobacter jejuni by IgG low- and high-concentration IgG patient serum*. Bacteria were incubated with 5% low- and high-concentration IgG patient serum, stained for IgG, IgM, C3, and C5b9 and subjected to flow cytometry. **a** Flow cytometry gating strategy. Opsonization with IgG (**b**), IgM (**c**), C3 (**d**), and C5b9 (**e**) was determined by flow cytometry. Measurement of geometric mean fluorescence intensity (MFI) was depicted in arbitrary units (AU (*n* = 6, mean ± standard error of the mean). A two-way paired Student *t* test was used for statistical analysis. *NS* not significant

To determine the capacity of the patient’s low- and high-concentration IgG serum to opsonize *C. jejuni*, we incubated the bacteria with 5% low- and high-concentration IgG serum followed by detection of IgG, IgM, complement factor 3 (C3), and membrane attack complex C5b9 opsonization by flow cytometry (Fig. 1a). Despite the large difference in IgG levels in the low- and high-concentration IgG samples, similar IgG opsonization of the bacterial surface (412 ± 73 and 462 ± 111, respectively) was detected, whereas IgM was absent (Fig. 1b, c). As functional read out for complement activation, we determined C3 and C5b-9 deposition on the bacterial surface. In accordance with IgG opsonization, no differences between low- and high-concentration IgG serum for C3 (11.043 ± 2.818 and 10.840 ± 3.183, respectively) or C5b-9 (1.011 ± 281 and 738 ± 214, respectively) were detected (Fig. 1d, e). These data indicated that the patient’s *C. jejuni* strain was recognized by IgG present in low- and high-concentration IgG serum, which was able to activate complement. Based on these data, we concluded that bactericidal activity of the low- and high-concentration IgG serum was similar.

### Augmented Complement Activation and Serum Killing of *Campylobacter Jejuni* with Serum IgM Supplementation

**Fig. 2:**
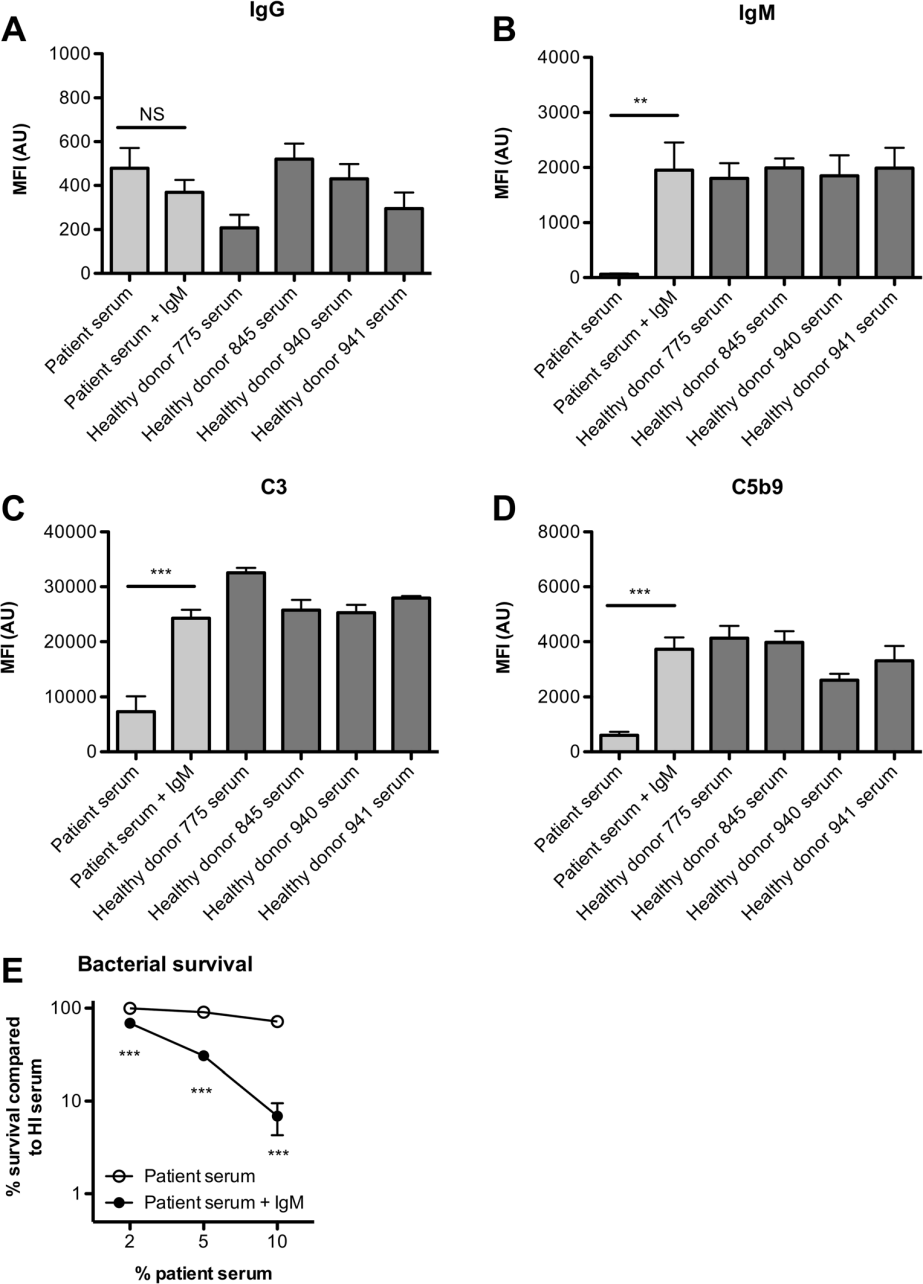
*Serum IgM augments complement deposition on the bacterial surface and serum killing of Campylobacter jejuni*. Bacteria were incubated with 5% patient serum with or without supplementation of IgM or 5% serum from healthy controls. Opsonization with IgG (**a**), IgM (**b**), C3 (**c**), and C5b9 (**d**) was determined by flow cytometry. Measurement of geometric mean fluorescence intensity (MFI) was depicted in arbitrary units (AU (*n* = 6, mean ± standard error of the mean). A one-way ANOVA with Dunnett’s Multiple Comparison Test was used for statistical analysis. *NS* not significant; **P < 0.01; ***P < 0.001. (**e**) Bacterial survival in 2, 5, or 10% patient serum with or without supplementation of IgM was determined after 30 min (*n* = 4, mean ± standard error of the mean). A two-way ANOVA with Bonferroni post hoc test was used for statistical analysis. ***P < 0.001

We determined whether IgM supplementation would improve complement activation and bactericidal activity against *C. jejuni*. For these experiments, a pool of low- and high-concentration IgG serum was used as patient serum and serum from four healthy individuals was included as normal reference. We measured IgG and IgM opsonization of the bacterial surface with 5% patient serum with or without IgM supplementation (5 mg/dL IgM in 5% serum, equivalent for 100 mg/dL IgM in normal serum). Opsonization with IgG was similar for 5% patient serum with or without IgM (369 ± 56 and 479 ± 92, respectively), which was within the normal range found for four healthy individuals (363 ± 70, range 208–521) (Fig. 2a). Opsonization with IgM was absent with patient serum and clearly present with patient serum supplemented with IgM (1.952 ± 502), which reached the normal range found for four healthy individuals (1.908 ± 48, range 1.803–1.993) (Fig. 2b). Although IgG in serum alone was able to activate complement as determined by C3 deposition on the bacterial surface, supplementation of IgM augmented C3 opsonization significantly (7.317 ± 2.805 to 24.295 ± 1.521), which reached levels close the levels found for four healthy individuals (27.877 ± 1.665, range 25.263–32.551) (Fig. 2c). Similarly, increased C5b9 deposition in the presence of IgM was observed (606 ± 123 to 3.736 ± 421), which was well within to the normal levels found for four healthy individuals (3.509 ± 347, range 2.612–4.133) (Fig. 2d). This increased complement activation was also represented in increased serum bactericidal activity because serum survival of *C. jejuni* in 2, 5, or 10% serum was significantly decreased with IgM supplementation (Fig. 2e).

## Discussion

Immunoglobulin replacement therapy was introduced by Colonel Ogden Bruton and applied to the first patients with agammaglobulinemia in 1952 [2] and remains the main therapy to date. Despite immunoglobulin therapy, agammaglobulinemia patients have increased infection rate and lowered life expectancy [4]. There have been no major advancements aside from subcutaneous immunoglobulin therapy in the early 1980s [14]. Recent advances in therapy for patients with agammaglobulinemia are mostly found in gene therapy [15, 16]. Although these methods show great potential, these are still far from being applied. Therefore, limiting infections and thereby increasing patient’s quality of life by supplementing IgM to IVIG would be an attractive alternative.

*Campylobacter* infections are frequently observed in patients with hypogammaglobulinemia, mainly causing gastrointestinal infections, despite IgGRT [17]. Besides frequent mucosal infections, *Campylobacter* species are also seen to cause bacteremia [18–20] and atypical infections in patient with agammaglobulinemia, including erysipelas-like skin lesions [19], arthritis [21], pericarditis [22], cellulitis [23], and spondylodiscitis (current report). Secretory IgA may act as an important local immune defense; however, there is no significant increase of *Campylobacter* infections in patients with selective IgA deficiency [17]. Therefore, concurrent IgM deficiency might decrease risk for bacterial infections because this class of antibody is especially good in complement fixation on bacterial pathogens.

Here, we show augmented serum bactericidal activity mediated by human serum IgM, which might increase protection against bacterial infections. Though C3 opsonization on bacteria may partly be explained by alternative complement pathway activation, our data indicate that IgM-mediated classical complement pathway activation is an important determinant. Our results support the use of IgM-enriched IgGRT in patients with IgG deficiency and concurrent IgM deficiency. For example, Borleffs and colleagues successfully treated two hypogammaglobulinemia patients with persistent gastro enteric *C. jejuni* infections [24]. Six dosages of Pentaglobin, an IgM-enriched intravenous IgG preparation containing 12% IgM and 12% IgA, at 3-week intervals increased bactericidal activity against *C. jejuni*, was well tolerated, and there were no culture-confirmed relapses of *C. jejuni* infections [24]. Pentaglobin was shown to contain both IgG and IgM antibodies recognizing *C. jejuni* outer membrane proteins [25]. In addition, two patients with relapsing *Campylobacter jejuni* infections were given fresh frozen plasma, which contains IgA and IgM, for 2 or 4 weeks (500 mL twice weekly) and resulted in cure in both patients [19]. Plasma infusion resulted in detectable serum IgA levels in two patients and detectable serum IgM levels in one patient [19].

The use of IgM-enriched intravenous IgG preparation Pentaglobin has been tested in different patient groups with variable results. Pentaglobin was shown to significantly reduce mortality in patients with sepsis [26, 27], but this was not corroborated by another study [28] or for critical illness polyneuropathy and/or myopathy patients with multi organ failure and sepsis [29]. Prophylactic use of IgM-enriched intravenous IgG preparation Pentaglobin has been tested in adults and pediatric patients after hematopoietic stem cell transplantation. In adults, a reduction in risk of dying of an infection after bone marrow transplant was observed [30]. However, in pediatric patients, no significant beneficial effects were observed [31]. A 2013 Cochrane database review concluded that the trials were too small and the totality of the evidence is still insufficient to support robust conclusions [32].

A beneficial effect of Pentoglobin in comparison to conventional intravenous IgGRT is its effective reduction of endotoxin [33, 34]. Direct effects on bacterial killing are also observed. Pentaglobin had a greater opsonic activity against *Pseudomonas aeruginosa*, *Klebsiella pneumoniae*, and *Escherichia coli*, in comparison to intravenous IgG Sandaglobulin [35]. Pentaglobin showed augmented in vitro killing of *Staphylococcus aureus*, *E. coli*, and *P. aeruginosa*, but slightly reduced killing *K. pneumoniae* and *Enterococcus faecium*, in comparison to intravenous IgG Intratect [36]. In a *S. aureus* mouse sepsis model, the number of bacteria in liver and kidney was significantly lower for animals receiving IgM-enriched IVIG Pentaglobin in comparison to animals receiving IVIG Intratect [36].

Recent advances in IgM-enriched immunoglobulin therapy, Trimodulin (BT-588, predecessor BT-086) containing more IgM (23%), in comparison to Pentaglobin (12%) are promising [37, 38], and a clinical trial in patients with severe pneumonia is underway [39]. Since IgM concentration present in Trimodulin is higher, we expect a strong additive effect on bacterial killing. It remains to be determined whether subcutaneous administration of IgM containing products offers sufficient bioavailability.

In conclusion, we show that supplementation of human serum IgM significantly contributes to complement activation on the bacterial surface and increases serum bactericidal activity. Therefore, supplementation of IgGRT with IgM may potentially offer greater protection against bacterial infections, which is also relevant in the context of rising antibiotic resistance. This hypothesis needs to be further tested in clinical studies.
